# Perceptions of Telehealth Services for Hearing Loss in South Africa’s Public Healthcare System

**DOI:** 10.3390/ijerph19137780

**Published:** 2022-06-24

**Authors:** Aaqilah Bhamjee, Talita le Roux, De Wet Swanepoel, Marien Alet Graham, Kurt Schlemmer, Faheema Mahomed-Asmail

**Affiliations:** 1 Department of Speech-Language Pathology and Audiology, University of Pretoria, Pretoria 0002, South Africa; bhamjeeaaqilah@gmail.com (A.B.); talita.leroux@up.ac.za (T.l.R.); dewet.swanepoel@up.ac.za (D.W.S.); surgeon@drkurtschlemmer.com (K.S.); 2 Ear Science Institute Australia, Subiaco, WA 6008, Australia; 3 Virtual Hearing Laboratory, Collaborative Initiative between University of Colorado and the University of Pretoria, Aurora, CO 10045, USA; 4 Department of Science, Mathematics and Technology Education, University of Pretoria, Pretoria 0002, South Africa; marien.graham@up.ac.za; 5 Department of ENT Head and Neck Surgery, University of Kwazulu Natal, Durban 4001, South Africa

**Keywords:** hearing loss, hearing healthcare, hearing resources, audiology, telehealth, South Africa, public healthcare

## Abstract

Telehealth promises increased access to hearing healthcare services, primarily in areas where hearing healthcare resources are limited, such as within the South African public healthcare system. Telehealth for hearing healthcare is especially important during the COVID-19 pandemic, where physical distancing has been essential. This study aimed to describe audiologists’ perceptions regarding telehealth services for hearing loss within South Africa’s public healthcare system. This study was divided into two phases. During Phase 1, 97 audiologists completed an electronic survey regarding their perceptions of telehealth for hearing loss within South African public sector hospitals. Synchronous virtual focus-group discussions were conducted during Phase 2. Results indicated that audiologists recognized telehealth services’ potential to improve hearing healthcare efficiency within the public sector, and most (84.1%) were willing to use it. However, telehealth’s actual uptake was low despite almost doubling during the COVID-19 pandemic. Prominent perceived barriers to telehealth were primarily related to hospital resources, including the unavailability of equipment for the remote hearing/specialized assessments, internet-related barriers, and limited IT infrastructure. An increased understanding of telehealth in South Africa’s public healthcare system will assist in identifying and in improving potential barriers to telehealth, including hospital resources and infrastructure.

## 1. Introduction

Globally, 1.5 billion people are estimated to have a hearing loss, with 430 million requiring rehabilitation services to address their disabling hearing loss [1]. By 2050, hearing loss prevalence will likely escalate to 2.5 billion, with 700 million people at a minimum requiring rehabilitation services [1]. At a micro-level, untreated hearing loss detrimentally impacts an individual’s quality of life, psychosocial health, social-communicative competence, economic independence, and one’s family and communication partners [2,3,4]. At a macro level, untreated hearing loss has implications for the country’s social and economic development resulting in an annual global loss of approximately US$ 1 trillion [1,5,6]. Hearing loss is therefore considered a significant global health concern, where prompt service provision to individuals affected by hearing loss is of utmost importance [7,8,9].

At present, there is a global shortage of hearing loss prevention and treatment programs [5]. Many individuals with hearing loss thus encounter challenges or barriers to accessing hearing healthcare services worldwide due to associated costs and a shortage of trained personnel [1,5,10,11,12,13,14,15]. There is also an unequal distribution of audiologists in high-income countries with it being exacerbated in low- to middle-income countries (LMIC) [1,5,11,13,14,16]. For instance, the majority (78%) of countries across Africa have less than one audiologist per million population, whereas more than half (52%) of the countries in Europe have more than ten audiologists per million population [1,16,17]. The poor audiologist-to-patient ratio, especially in LMICs, negatively impacts patients’ accessibility to specialized hearing healthcare, including hearing loss diagnosis and management [1]. Consequently, audiology services cannot adequately manage the global burden of hearing loss due to these ingrained barriers toward hearing healthcare [18]. It is thus critical for hearing loss identification and treatment to be addressed from a different stance, using innovative solutions [15].

Hearing healthcare service delivery via telehealth is a promising way to improve access and affordability of hearing healthcare services, and it is recommended as a priority by the recent World Report on Hearing [1,11,14,18,19]. Telehealth refers to the use of information and communication technology in healthcare, and it allows for hearing healthcare accessibility to underserved populations across the globe [11]. The COVID-19 pandemic has further intensified the importance of telehealth and remote services due to physical distancing and precautionary measures required during service provision [10,20]. In this scenario, where outpatient services are interrupted, telehealth may help to provide immediate access to rehabilitation services, allow for continuous monitoring of patients and improve patients’ health outcomes and quality of life [21]. Furthermore, there has been a global change of attitude and improvement toward telehealth for hearing loss due to the pandemic [22]. Low- and no-touch service delivery models are crucial for patients requiring hearing healthcare as many of these patients are at increased risk for COVID-19 mortality and morbidity due to their age since most patients requiring these services are above 65 years [20]. With the possibility of hearing care being provided to approximately 95% of hearing-impaired adults in low- and no-touch service delivery models [20,23], the use of telehealth for audiology service delivery during the COVID-19 pandemic and beyond is important. Therefore, a well-thought-out strategy to include telehealth and remote services can enable improved access to hearing healthcare and patient support, decrease costs related to hearing healthcare, and decentralize access [10,24].

Despite increasing evidence promoting the use of telehealth in the field of audiology, its clinical uptake has been slow, and there are limited reports of its utilization in everyday clinical practice [10,25]. The successful implementation of telehealth in hearing healthcare largely depends on the audiologists and their role in providing telehealth services [26]. Studies have been conducted on the attitudes of audiologists concerning telehealth prior to the COVID-19 pandemic [27,28] and also during the COVID-19 pandemic [22,29,30,31]. While two of these studies were international studies [22,27], which included participants from South Africa as part of their samples (16.8% [22] and 39.8% [27] of South African participants), the results did not indicate whether the audiologists represented private or public healthcare systems. The majority (84%) of the South African population relies solely on public sector services in contrast to a marginal 16% of the population served by South Africa’s self-funded private sector [32,33]. This inequality in South African healthcare is further exacerbated by patients’ unequal accessibility and their outcomes across the urban and rural public healthcare sectors [33]. Therefore, audiologists’ perceptions of telehealth services within the South African public healthcare system across urban and rural areas must be recognized.

In line with the 2021 World Report on Hearing, which has prioritized telehealth to make hearing care more accessible, understanding the perceptions of hearing healthcare professionals can support guidance for future implementations of telehealth services [1]. Therefore, the current study aimed to describe audiologists’ perceptions of telehealth services for hearing loss in South Africa’s public healthcare system

## 2. Materials and Methods

Research and ethical approval for this study were obtained from the Research Ethics Committee of the Faculty of Humanities, University of Pretoria, South Africa (HUM005/1019) before any participants were recruited for this study.

A survey-based cross sectional study with a subsequent thematic qualitative analysis, applying triangulation, was employed using a two-phase process involving an online survey in Phase 1 followed by focus group discussions in Phase 2. This research design facilitated a deeper and more rigorous understanding of audiologists’ perceptions of telehealth services in South Africa’s public healthcare sector by combining both quantitative (explorative) and qualitative (deepening) data.

### 2.1. Study Population

In 2020, 182 public hospitals across South Africa, including district, regional, provincial tertiary, central, and specialized hospitals, had audiology departments, with approximately 380 audiologists employed across these hospitals [34]. All qualified audiologists employed at these public hospitals were considered eligible participants for this study. More than one audiologist per hospital was allowed to participate in this study and to provide their perspectives on telehealth service delivery for hearing loss within the public sector.

### 2.2. Materials for Data Collection

In Phase 1, data were collected by means of a self-administered survey explicitly developed for the purpose of this study. First, a pilot study involving five volunteering audiologists employed within South African public sector hospitals was conducted to establish the validity of the newly developed survey. The expert panel had some minor recommendations (sentence reconstruction for questions eight and nine for clarity purposes), which were incorporated into the survey. An item content validity index (I-CVI) was computed, which provided the proportion of experts agreeing on an item [35]. An acceptable I-CVI value is 1 when the expert panel consists of three to five experts [36]. Content validity was established since the I-CVI equaled 1 for each item in the newly developed survey.

The survey was used to obtain information concerning audiologists’ perceptions of telehealth services for hearing loss in South Africa’s public healthcare system. It included nine closed-ended questions, with one open-ended question included (Supplementary Materials S1). The survey comprised three sub-sections; (i) audiologists’ demographics (two questions), (ii) hospital’s demographics (one question), and (iii) audiologists’ perceptions of telehealth services in South Africa’s public healthcare system (seven questions). Four questions (questions five, six, seven, and nine) included in the survey were adapted from an international survey [22]. Question nine of the survey (potential barriers toward telehealth service delivery) consisted of 22 statements, and audiologists rated their responses on a 5-point Likert scale ranging from 1 (strongly disagree) to 5 (strongly agree). Response ratings of ‘strongly agree’ and ‘agree’ were grouped together as an ‘agree’ response, response ratings of ‘strongly disagree’ and ‘disagree’ were grouped together as a ‘disagree’ response, response ratings for ‘either no comment or statement not applicable to respondent’ are indicated as a neutral response. Incomplete or no responses to questions in the survey were processed as missing values.

In Phase 2 of the study, qualitative data were collected to expand and to substantiate findings from the survey. Two virtual focus group discussions were conducted, consisting of four and five audiologists in each respective focus group. A semi-structured focus group guide was compiled, which provided structure and an agenda for audiologists whilst engaged in conversation (Supplementary Materials S2). The questions were formulated to probe audiologists’ perspectives on telehealth, the use of telehealth practices within the public sector workplace, the patients they felt would benefit from telehealth services, their perceived advantages or benefits of telehealth, as well as their perceived concerns regarding telehealth.

### 2.3. Procedures

In Phase 1 of this study, all 380 audiologists employed within public sector hospitals (district, regional, tertiary, central, and specialized hospitals) in 2020 were invited to participate in the survey via email. The audiologists were sent via email the information letter detailing the nature of the study and their level of involvement, along with the website link to the online survey utilized for this study.

Once audiologists clicked on the website link, an informed consent page opened, and only audiologists who electronically consented to participation proceeded to the survey. Data for Phase 1 of the study were collected over a one-month period during the COVID-19 pandemic when the country was under level one lockdown. The response rate was 25.5% in a purposive sample of 97 audiologists who responded to the survey, nine of whom participated in Phase 2 of the study.

At the end of the survey, participating audiologists were asked whether they would be interested in participating in the second phase of this study, namely synchronous, virtual focus group discussions. A total of 45 audiologists expressed interest in participating in Phase 2, and they were subsequently sent via email the information letter detailing the nature of Phase 2 and the website link to provide informed consent and indicate their preferred availability for the focus groups. Of the 45 interested audiologists, nine audiologists were available and provided consent allowing for two focus groups. The focus group discussions were conducted and recorded using a web-based virtual communication platform (Microsoft Teams). The primary researcher (A.B) administered the role of the facilitator of each focus group discussion. Each focus group discussion was recorded and transcribed following the discussion for data analyses.

### 2.4. Data Analysis

The quantitative data of Phase 1 (close-ended questions of the survey) were captured in Microsoft Excel (2017), and Statistical Package for Social Sciences SPSS (Version 26) was used to analyze the data. Cronbach’s Alpha test was used to calculate internal consistency for the scale used for Question 9 (potential barriers toward telehealth service delivery) in the questionnaire. An exploratory factor analysis (EFA) was subsequently conducted on Question 9 with the Kaiser–Meyer–Olkin (KMO) and Bartlett’s test of sphericity considered to see whether the data is suitable for dimension reduction. The KMO value of 0.759 is middling (i.e., acceptable) [37], indicating that the data is suitable for factor analysis. The p-value of Bartlett’s test of sphericity is less than 0.05 (*p* < 0.001), indicating that there is evidence that dimension reduction can be done. After conducting the EFA (using Promax rotation to allow for constructs to be correlated, as was evident from the Component Correlation Matrix) and dropping items with low loadings on factors/constructs, a summary of the final set of constructs, item statements, and Cronbach’s Alpha values for Question 9 can be found in Table S1. It is generally accepted that a Cronbach’s alpha value of 0.70 or greater is a reasonable indication of the reliability of a scale [38]. However, some authors have indicated that the alpha value of 0.60 is also considered acceptable [39,40,41]. Thus, since all Cronbach’s alpha values ranged from 0.690 to 0.927, the instrument’s reliability was established. Furthermore, using the formula of Bonett (2002) for constructs of 3 items (which is the case for this questionnaire), the minimum sample size required for Cronbach’s alpha is 31 observations [42], which we exceeded.

**Figure 1:**
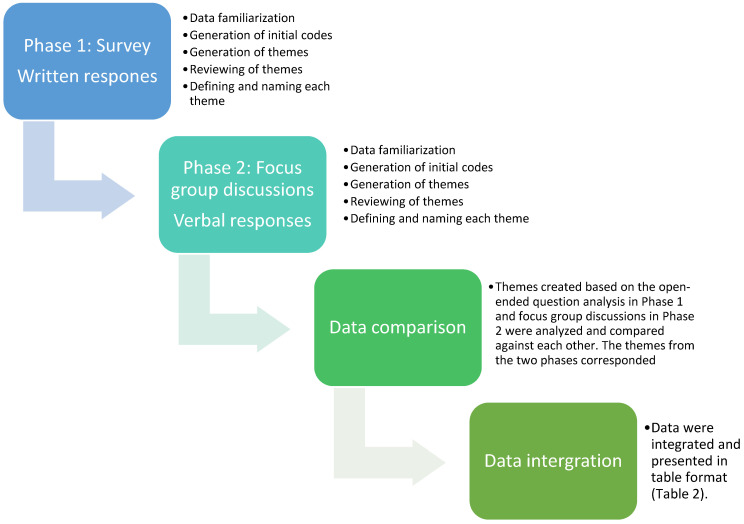
Process flow chart of thematic analysis.

Qualitative content analysis of the responses to both the open-ended survey question (Question 10) and the focus group discussions was conducted utilizing the conventional approach as identified by Braun and Clark [43] (Figure 1).

## 3. Results

### 3.1. Quantitative Results

Of the 97 participating audiologists, 49 (50.5%) worked at district level, 17 (17.5%) at regional, 23 (23.7%) at tertiary, four (4.1%) at central, and four (4.1%) at specialized levels of care public sector hospitals. The majority of the participating audiologists (90.7%, *n* = 88) held a bachelor’s degree in audiology, or a dual (speech-language pathology and audiology) degree, whilst nine (9.3%) of the audiologists held a master’s degree.

**Figure 2:**
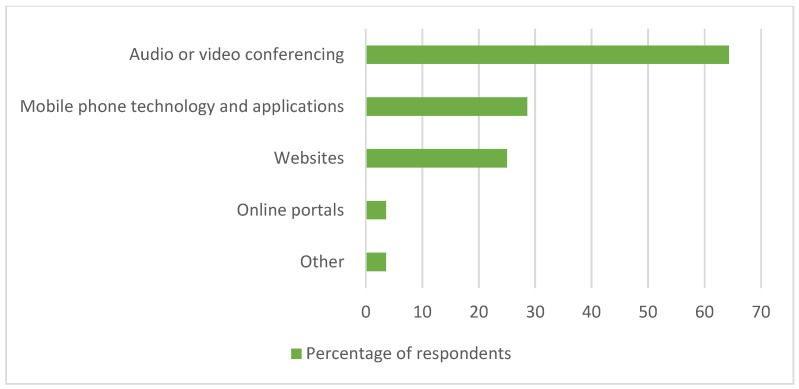
Types of telehealth services used in audiology departments within South African public sector hospitals as perceived by audiologists, indicated in terms of % (*n* = 28).

Almost one-fifth of the audiologists indicated that their hospital presently used telehealth services for hearing healthcare (19.6%, *n* = 19), and almost one-tenth were unsure (9.3%, *n* = 9) of whether they were using telehealth or not. Both these groups went on to then select the applicable type(s) of telehealth options used from a list provided, although the questionnaire stated that only those that responded ‘yes’ to the question of whether their workplace currently used telehealth should go on to list the types of telehealth they were using. The most frequently reported type was audio or video conferencing (64.3%, *n* = 18/28), followed by mobile phone technology and applications (28.6%, *n* = 8/28), and websites (25.0%, *n* = 7/28) (Figure 2).

Of the audiologists who perceived that their hospital was not using any telehealth services (71.1%, *n* = 69/97), 84.1% (*n* = 53/63) indicated that they would be willing to use it (Table 1).

**Figure 3:**
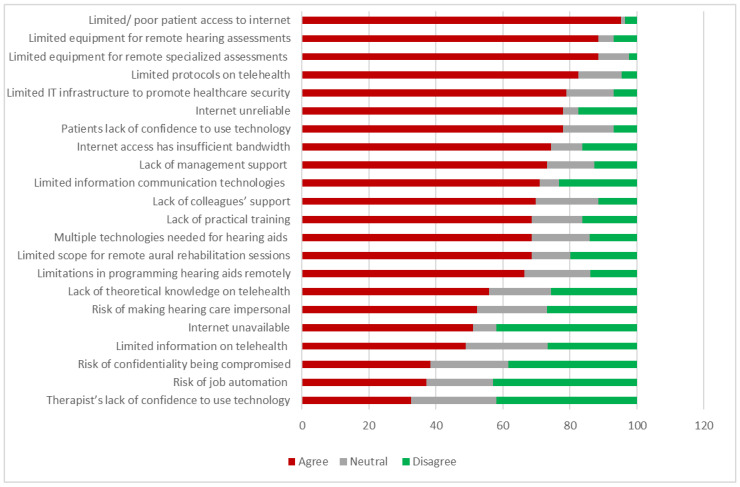
Perceived barriers toward telehealth service delivery within South African public sector hospitals, indicated in terms of % (*n* = 86).

When asked to indicate their level of agreement on potential barriers toward service delivery, poor patient access to the internet was the most prominent perceived barrier reported by the audiologists (95.3%, *n* = 82/86) (Figure 3). Furthermore, audiologists also indicated that internet access at their hospital was unreliable (77.9%, *n* = 67/86) and that internet access at their hospital did not have enough bandwidth 74.4% (*n* = 64/86) (Figure 3).

Items are shown in order of most significant barrier and are slightly abbreviated from the phrases used in the survey; see Supplementary Materials S1 for the full text of survey questions and answers.

### 3.2. Qualitative Findings

For the focus group discussions, four of the audiologists attended the first focus group discussion, and five attended the second. Four audiologists worked in tertiary-, three in district-, one in central-, and one in regional-level hospitals. Seven of the audiologists were production-level therapists, and two audiologists were the heads of their departments.

Following inductive thematic content analysis of the two focus group discussions, it became apparent that the identified focus group themes corresponded to the themes identified in the open-ended question analysis: (1) clinical practices, (2) hearing healthcare resources, (3) patient restrictions impacting hearing healthcare, and (4) perceived benefits of telehealth. Therefore, the results from the open-ended question in the survey and focus group responses were integrated (Table 2).

## 4. Discussion

This study investigated audiologists’ perceptions of telehealth for hearing healthcare services within the South African public healthcare system, and it found low use of telehealth services before the COVID-19 pandemic. The use of these services increased during the COVID-19 pandemic. Hospital- and patient-related barriers to telehealth were mentioned by audiologists, and the most prominent perceived benefit of telehealth services was increased accessibility to hearing healthcare services.

Telehealth services in hearing healthcare increased as a result of the COVID-19 pandemic, from only 7.2% reporting its use before COVID-19 to almost 19.6% using it during the pandemic. Similarly, other studies have previously reported an increase in telehealth to deliver audiology services during the COVID-19 pandemic [22,31]. Most (84.1%) audiologists who indicated that their hospitals were not providing telehealth services were willing to use it. These findings are in line with previous study findings, which demonstrated that audiologists are cognizant of telehealth’s ability to improve audiological service delivery in general [27,28] and during the COVID-19 pandemic specifically [22,30,31]. Similarly, the qualitative data obtained on the perceived benefits of telehealth in this study indicated that audiologists believe telehealth has potentially positive outcomes for patient travel and travel costs, increased patient accessibility to audiological service delivery, and convenience for audiologists as well as patients. These reported positive telehealth enablers are comparable to the data of Singh et al. (2014), Saunders et al. (2021), and Eikelboom et al. (2021). Furthermore, in this study, audiologists perceived telehealth as a valuable tool during the COVID-19 pandemic for infection control purposes and to minimize the risk of contact.

Despite a general recognition of the potential telehealth service delivery holds in the field of hearing healthcare, the actual perceived use of telehealth during the COVID-19 pandemic in the current study was significantly lower than that reported in previous studies. The reported telehealth usage of an international survey spanning 44 countries reported that 61.9% of audiologists in their study used telehealth services during the pandemic [22]. When looking specifically at an upper-income country such as the UK [31], the usage percentage increases to 98%. The reason for this vast discrepancy may be due to the scarce healthcare resources available in South African public healthcare systems.

Identifying perceived barriers toward telehealth is important to understand the reason behind the low uptake within South Africa’s public healthcare system and address the concerns accordingly. In line with the findings from Eikelboom et al. (2021), the least significant barriers perceived by audiologists in this study were related to the audiologists themselves (audiologists’ confidence and job security). More commonly perceived barriers included training, multiple technologies needed for hearing aids, limited scope for remote aural rehabilitation sessions, and limitations in programming hearing aids remotely. The most significant perceived barriers were external to the audiologists and involved patient-related aspects such as limited/poor patient access to the internet and patients’ lack of confidence to use technology. It also included barriers related to resources, such as the unavailability of equipment for remote hearing and specialized assessments, internet-related barriers, limited IT infrastructure, and limited protocols for telehealth. The majority of these audiologist-perceived barriers toward telehealth were reiterated by audiologists in their open-ended responses and during the focus group discussions, and they were also recently mentioned in other studies as well [22,27,29,30,31,44].

The South African health system is dichotomized [45]. Most South Africans rely on public healthcare sector services, where hearing healthcare is unfortunately not prioritized because South Africa faces a quadruple burden of disease [32,33,45]. Furthermore, a recent national study on hearing healthcare in South Africa concluded that hearing healthcare resources and services are perceived by audiologists as lacking within the South African public healthcare system [46]. Therefore, it is not surprising that during open-ended responses and focus group discussions, audiologists in the current study indicated that limitations and challenges within the public healthcare system would make one question the feasibility and the viability of telehealth within this setting. In part, it also justifies why 74.1% of audiologists in the current study indicated that they would be willing to use telehealth but did not have the resources to do so. A shortage of healthcare resources was further emphasized by the qualitative data obtained. As reported in this study, many public sector hospitals in South Africa have limited access to IT support and internet, equipment, infrastructure, and human resources, all of which are crucial for implementing telehealth practices. Previous studies within the South African public healthcare system have revealed a shortage of audiologists as well as infrastructural, equipment, and financial challenges within public sector audiology departments [46,47]. Ring-fenced funding to support prioritized initiatives to improve the accessibility of hearing healthcare services such as telehealth within South Africa’s public healthcare system is therefore warranted.

Another prominent perceived barrier to telehealth was the restrictions faced by patients attending these public sector hospitals, as indicated by both the qualitative and the quantitative data. In line with this study’s findings, uncertainties on the part of audiologists on whether their patients would cope with the technology needed for telehealth were also reported in other studies [22,26,31,44,48]. A recent study investigating the effect of self-perceived digital proficiency on the uptake of hearing healthcare services demonstrated that patients who sought hearing healthcare services at the Hearing Research Clinic Non-Profit Company were proficient in using mobile devices or computers [49]. However, this study included participants with internet access and who were financially able to pay for hearing health services [49] as opposed to patients attending public sector hospitals and whose socio-economic status may be different. Many of these patients come from poor socio-economic backgrounds and deep rural areas, where poverty, unemployment, insufficient levels of education, illiteracy [50], and the affordability of data costs could negatively impact their access to the technology required for telehealth services and their confidence to use this technology. Therefore, despite the global increase in mobile penetration and roughly one-third of the South African population utilizing smartphones [51,52], further research into smartphone ownership/accessibility amongst patients receiving South African public sector services will provide more insight into the possibilities for telehealth and particularly mHealth within this sector. Furthermore, recent research has emphasized the crucial role audiologists need to play to promote technological literacy to help patients communicate on virtual platforms in response to their new hearing needs when using telehealth [53].

Telehealth services are a promising tool for improving the accessibility of hearing healthcare services [54,55], especially within the resource-strained South African public healthcare system [46,47]. The COVID-19 pandemic has further highlighted its usefulness in increasing accessibility to audiological services to patients, even more so when lockdown measures are in place, thereby necessitating remote service options [10,56]. Ultimately, the provision of telehealth for audiological service delivery depends on multiple factors such as the audiologists themselves, infrastructure, access to high-speed internet, cost, and socio-cultural aspects [26]. Data obtained from this study can guide national policy toward improving hearing healthcare resources within South Africa at a national level, especially within the public healthcare system. This will ensure that the public sector is well-resourced to competently deliver hearing healthcare services, including telehealth services, to all patients in need of these services.

Limitations of this study include a relatively low response rate (approximately 1 in 4). However, recently [57], and especially during the COVID-19 pandemic [58], response rates to online surveys have declined dramatically due to the rise of online surveys, survey-based studies, and online information requests, and a greater awareness of privacy issues. These issues all lead to a phenomenon known as survey fatigue resulting in decreased response rates. A future recommendation would be to explore a more direct in-person approach (i.e., delivering surveys in person and collecting them); however, these options were limited during the COVID-19 pandemic, when data collection for this study took place. Furthermore, it is worth mentioning that the number of focus group discussions and the sample size for Phase 2 of the study were in fact sufficient in order to reach data saturation [59,60]. Even though this sample represents the perceptions of audiologists across the different levels of care within the South African public healthcare system (district, regional, tertiary, central, and specialized), it does not represent the national population of audiologists within the public sector. It should also be noted that only audiologists familiar with telehealth services can accurately opine on the value of telehealth services to their patients. While this study did not focus on only audiologists familiar with telehealth services, future research should focus on this to determine its value to patients within South African public sector hospitals. Nevertheless, this was the first study to exclusively investigate the perceptions of telehealth services for hearing loss within the South African public healthcare system.

## 5. Conclusions

The majority of public sector audiologists reported a willingness to use telehealth services. They were also aware of its potential to improve hearing healthcare accessibility and efficiency within the public sector, especially during the COVID-19 pandemic. However, the uptake of telehealth services was poor despite more than doubling during the COVID-19 pandemic. Prominent barriers that were primarily identified related to limitations in hospitals’ resources and patient-related barriers, including patient restrictions that impacted their means to access required telehealth technology and a lack of confidence that patients were in a favorable position to receive telehealth services.

## Figures and Tables

**Table 1:** Audiologists’ perceptions of telehealth practices within their hospital setting (*n* = 97).

**Telehealth use by audiologists**	**% (*n*)**
Unaware of what telehealth entails	8.2 (8)
Not willing to use telehealth	2.1 (2)
Willing, but no resources	74.2 (72)
Using telehealth, and it is effective	8.2 (8)
Other	7.2 (7)
**Use of telehealth services pre-COVID-19 setting**	**% (*n*)**
No	87.6 (85)
Yes	7.2 (7)
Unsure	5.2 (5)
**Use of telehealth services currently (during COVID-19 setting)**	**% (*n*)**
No	71.1 (69)
Yes	19.6 (19)
Unsure	9.3 (9)
Willingness to use telehealth services *	**% (*n*)**
No	6.4 (4)
Yes	84.1 (53)
Unsure	9.5 (6)

* *n* value is altered since this question was only applicable to the 69 audiologists who previously indicated that they did not use telehealth services (missing data for six audiologists).

**Table 2:** Themes, Categories, Descriptions, and Illustrative Examples of Focus Group Discussion on Perceptions of Telehealth Services for Hearing Loss in the Public Healthcare System.

Themes and Sub-Themes	Description	Illustrative Examples
1. Clinical practices		
Limitations of remote hearing healthcare	Not applicable to all populations; hearing loss and language barriers affect telehealth communication.	‘Certain information is better understood by physical contact rather than electronic contact. Patients with multiple disabilities may struggle even further.’ ‘Hearing loss and language barriers through telephones and other technology can hamper ability to communicate.’
	Impersonal nature; negative impact on patient relationship.	‘In a field like Audiology, where patients’ main difficulty is hearing, it may be difficult to impossible to get messages across to them effectively.’
	Quality of service and audiologists’ preferences for face-to-face consultations.	‘For me, even if the infrastructure is in place, I am not really in favour of telehealth. I prefer to render quality face to face interaction types of services and feel that this can’t be replaced by telehealth.’
Limitations of South Africa’s public healthcare setting	Limitations and challenges in the healthcare system question the feasibility and the viability of telehealth within this setting.	‘Public health institutions, especially at primary healthcare level and those in rural areas are also too poorly equipped to be able to effectively provide these services. Telehealth in South Africa’s public health system thus faces large barriers to be successfully implemented at this time.’
Policy and protocol	Lack of policies, protocols, and guidelines to guide the use of telehealth.	‘My concern is with the record-keeping. When patients come in for an appointment, I know the procedure. With telehealth, there are no clear guidelines. Also, there are no protocols to distinguish when to use what form of telehealth and no guidelines.’
Knowledge and training	Increased telehealth training needed for audiologists; knowledge and training to hospital management and policymakers; promotion and awareness of telehealth services.	‘The telehealth system is grossly underdeveloped and requires additional training by all healthcare workers.’ ‘If leaders and those in power can be educated about telehealth, it can be easier to have access to equipment necessary for telehealth.’ ‘There is a lack of public awareness and understanding of the potential benefits of telehealth’.
2. Hearing healthcare resources		
Information systems and technology	Lack of IT and software support required, including limited or no access to the internet at hospitals.	‘Telehealth services are a great challenge in low resourced hospitals or rural communities. Thus, access to reliable internet and coverage remains the greatest barrier to achieving telehealth services.’
Equipment and infrastructure	Lack of equipment and infrastructural resources.	‘Unfortunately, the public health system hasn’t really invested in procurement of equipment which is telehealth compatible.’
Human resources	Shortage of audiology staff in many of the South African public sector hospitals.	‘In government, we know that the organogram is constantly changing, posts are frozen if therapists leave, and new therapists are seldom hired, so the staffing, in addition to the hospital’s infrastructure, is a big challenge to telehealth.’
3. Patient restrictions impacting hearing healthcare		
Financial resources	Many patients are unable to afford the resources required to access telehealth services.	‘South Africa’s public healthcare system is largely used by people from poor socio-economic backgrounds, and thus unable to access the technology required to receive telehealth services.’
Education	High percentage of uneducated and/ or illiterate patients receive public healthcare sector services.	‘A majority of our patients are not educated, thus making the use of teleaudiology almost impossible.’ ‘Most patients are from very poor backgrounds and are illiterate.’
Employment	Employment status impacts patients’ ability to access telehealth services.	‘Most patients served in public are unemployed.’
4. Perceived benefits of telehealth		
Accessibility to services	Increased accessibility of cost-efficient and time-efficient audiology services to a broader population,-eliminating transport and travelling costs; the convenience of telehealth for patients and audiologists.	‘I believe telehealth has the potential to bridge the access gap for patients, particularly those living in rural areas who have limited access to hearing health professionals.’ ‘For me, the biggest advantage is the convenience. The convenience for ourselves as well as our patients. It means that reduces their travel time and a whole lot of anxiety.’
COVID-19 pandemic	Current COVID-19 pandemic highlights the usefulness and the value of telehealth services as a means of infection control and minimizing the risk of contact.	‘Telehealth services is a viable solution considering the pandemic’ ‘It would assist a lot, especially during this pandemic (to reduce infection).’
Potential and willingness to use	Recognition of potential use of telehealth and willingness to use it; the need to adapt and to modify telehealth practices according to available resources.	‘Telehealth requires adaption. Many might display hesitation, but we need to keep up and ‘go with the flow’ in an ethical way using evidence-based practices for telehealth.’ ‘I believe that telehealth has the ability to work well in the public sector.’

## Data Availability

The data supporting this study’s findings are available on request from the corresponding author (F.M.-A.). The data are not publicly available due to their containing information that could compromise the privacy of research participants.

## Supplementary Materials

The following supporting information can be downloaded at: https://www.mdpi.com/article/10.3390/ijerph19137780/s1, Table S1: Constructs, Items per Construct and the Corresponding Cronbach’s Alpha Values.
