# Supplementary material for: Perceptions of Telehealth Services for Hearing Loss in South Africa’s Public Healthcare System
Source: Int J Environ Res Public Health. 2022 Jun 24;19(13):7780. doi: 10.3390/ijerph19137780 (PMC9265507; doi:10.3390/ijerph19137780)
Supplement: Supplementary file 1 [file ijerph-19-07780-s001.zip › Supplementary S1.pdf]

Perceptions of telehealth services for hearing loss in South Africa's public healthcare system

Section A: Information about you as an audiologist employed within a public sector workplace

Name of the Audiologist: ____________________________
(Please note that the Audiologist's name is needed for data tracking purposes and will be omitted during data analysis)

1. Your highest qualification level obtained can best be described as:

Bachelor's Degree in Audiology	
Master's Degree in Audiology	
Doctoral Degree in Audiology	
Other (please specify)<br>……	

2. Years of experience as an Audiologist:

Less than 2 years	
2 to 5 years	
5 years, 1 day to 10 years	
10 years, 1 day to 15 years	
More than 15 years	

Section B: Information about your current workplace setting

Name of the workplace site: ____________________________
(Please note that the site's name is needed for data tracking purposes and will be omitted during data analysis)

3. Level of care of your current workplace:

District	
Regional	
Tertiary	
Central	
Specialized Hospitals (e.g., Specialized TB hospitals)	

Section C: Telehealth

Given the current COVID-19 pandemic and its impact on audiological service delivery, this section aims to find out more about your perceptions of telehealth practices in your workplace site.

The World Health Organization (WHO) defines telehealth as "the delivery of healthcare services, where patients and providers are separated by distance. Telehealth uses information and communications technology for the exchange of information for the diagnosis and treatment of diseases and injuries, research and evaluation, and for the continuing education of health professionals."

4. What are your perceptions of telehealth practice? Please select one option

I am unaware of what telehealth entails	
I am not willing to use telehealth	
I am willing to use telehealth; however, I do not have the resources to do so	
I am using telehealth, and it is effective	
Other (please specify) ………	

5. Based on your perceptions, has your workplace site made use of telehealth services prior to the COVID-19 pandemic? *

No	
Yes	
Unsure	

6. Based on your perceptions, is your workplace site currently using any telehealth services? *

No	
Yes	
Unsure	

7. Since you answered "yes" to the previous question, please tick all the telehealth services you perceive your workplace site to be currently using? *

Websites	
Online portals (hospital-run patient portals offering online visits)	
Audio or video conferencing (zoom, WhatsApp calls, google meets)	
Mobile phone technology and applications (smartphone hearing screening, pure-tone audiometry, diagnostic hearing assessments, video otoscopy, remote cochlear implant or hearing aid programming, etc.)	
DVD's and CD's	
Other (please specify) ………	

8. Since you answered "no" to the previous question, would you be willing to use telehealth services within your workplace site?

No	
Yes	
Unsure	

9. Based on your perceptions, please indicate your level of agreement on the following potential barriers towards telehealth service delivery. *

Potential barriers	Strongly disagree	Disagree	Neutral (either you have no comment or the statement is not applicable to you)	Agree	Strongly agree
Internet access at my site is unreliable					
Limited information available on telehealth in audiology					
Lack of practical training on telehealth					
Multiple technologies required for each of the different hearing aid manufacturers					
There is a risk of confidentiality being compromised					
Limited scope for hearing aids to be programmed/ adjusted remotely					
Internet access at my site doesn't have enough bandwidth					
Lack of scope from senior management in introducing telehealth					
Limited equipment for conducting hearing assessments remotely					
Limited/ poor patient access to internet					
There is a lack of IT infrastructure to promote healthcare security and safeguard against cyber-attacks, phishing attacks etc.					
The risk of making hearing care impersonal					
Internet access at my site is unavailable					
Limited access to information communication technologies at my site (such as computers, tablets, video equipment)					
My lack of confidence using telehealth technology					
Patients lack of confidence using technology					

**Limited equipment for specialized assessments remotely, such as auditory evoked potentials, vemps, etc.**					
**Limited protocols available on telehealth in audiology**					
**Limited scope for aural rehabilitation to be performed remotely**					
**The risk that my job may be automated**					
**Lack of scope from colleagues in introducing telehealth**					
**Lack of theoretical knowledge on telehealth**					
**Other (please specify)** ………………………………………………………………………… ………………………………………………………………………… ………………………………………………………………………… …………………………………………………………………………					

10. Please describe your overall perceptions of telehealth services in South Africa's public health care system

____
____
____
____
____
____
____
____
____
____
____
____
____

Would you be interested in participating in a follow-up virtual focus group discussion centered on Audiologists' perceptions of telehealth services for hearing loss in South Africa's public healthcare system? Each focus group participant will receive a Takealot voucher to the value of R150.00

Yes, I am interested	
No, I am not interested	

* Questions adapted from the "International survey of audiologists during the COVID-19 pandemic: use of and attitudes to telehealth." (Eikelboom et al., 2021).

THANK YOU for taking the time to answer these questions
