# Supplementary material for: Perceptions of Telehealth Services for Hearing Loss in South Africa’s Public Healthcare System
Source: Int J Environ Res Public Health. 2022 Jun 24;19(13):7780. doi: 10.3390/ijerph19137780 (PMC9265507; doi:10.3390/ijerph19137780)
Supplement: Supplementary file 1 [file ijerph-19-07780-s001.zip › Supplementary S2.pdf]

Focus group interview guide.

Welcoming

Good evening and welcome to our session. Thank you for taking the time to join us to talk about telehealth services in South Africa's public health care system. My name is Aaqilah Bhamjee, and I am a postgraduate student at the University of Pretoria. I will be the moderator for this evening's session.

Overview of the topic

This session aims to expand on the information gained from the survey on *Audiologists' perceptions of telehealth services within the South African public healthcare system* you recently completed. In doing so, we would like to find out more about your understanding and perceptions of telehealth services. You were invited because you are an Audiologist employed within a public sector hospital and are familiar with the setting. Also, you participated in phase one of the study (electronic survey), during which time you indicated that you would be willing to participate in the follow-up focus group discussion.

Ground rules and guidelines

I want to highlight some guidelines and ground rules for the discussion today.

- The focus group will last between 30-45 minutes.
- I have several questions for you. After each question, you will discuss the question with each other. To ensure that we stick to the time limit, I will apologize in advance if I may have to interrupt your discussion and move you on to the next question. The priority is to ensure that we get a chance to hear about all the questions on the agenda within our designated time allocation.
- Everyone should participate, and only one person should talk at a time. It is important for us to hear all participant's perspectives and opinions.
- There are no right or wrong answers to questions – just ideas, experiences, perceptions, and opinions, which are all valuable. Please feel free to share your point of view, even if it differs from what others have said.
- You have probably noticed that this session is being recorded. This is to ensure that no comments are missed and to assist during the analysis process.
- Try to stay with the group and avoid having side conversations.

- Even though we will be on a first-name basis throughout the discussion, no names will be utilized in the research report. Therefore you may be assured of complete confidentiality.

Well, let's begin. Let us first find out more about each other. Can everyone can introduce themselves by telling us your name and the hospital where you work.

Now that we are all acquainted with each other, we can begin with the discussion.

Opening question

1. Let us hear all the things that come to mind when you think about telehealth.

Note: If a group is sustaining its own discussion, there will not be a need to probe.
If it is not sustaining its discussion, the moderator will need to probe the discussion.

Probes may include:

Free probes:
'What else?'
'Does anyone have a different thought?'

Specific probes:
'Uses of telehealth: prevention; screening; assessment; treatment; hearing aid/ assistive technology; counselling; follow-ups; aural re(habilitation); equipment check'.
'Modes of telehealth: telephone; email; web-based information; streaming video/ chat room; video conferencing etc.'
'Resources required (equipment, staff, infrastructure, and finances)

Approximate time allocation for this question: 5 minutes

2. Given the current COVID-19 pandemic, what are your thoughts on using telehealth practices within your public sector workplace?

Probes may include:

Free probes:
'What else?'
'Does anyone have a different thought?'

Specific probes:
'Uses of telehealth: prevention; screening; assessment; treatment; hearing aid/ assistive technology; counselling; follow-ups; aural re(habilitation); equipment check'.
'Modes of telehealth: telephone; email; web-based information; streaming video/ chat room; video conferencing etc.'
'Resources required (equipment, staff, infrastructure, and finances)
'Who can give examples of telehealth services used in the public sector?'
'Internet technology and systems'
'Patient factors such as lack of infrastructure and technology.'
'Reliability of results'
'Confidentiality of information
'Level of training and knowledge of telehealth.'
'Protocols and policies for telehealth.'
'Concerns about malpractice.'
'Quality of telehealth versus face-to-face interaction.'

Approximate time allocation for this question: 5 minutes

3. Which patients do you feel would benefit from telehealth services?

Probes may include:

Free probes:
'Any other patients?'

Specific probes:
'Pediatric patients'
'Adult patients.'
'Geriatric patients'
'Aural (re)habilitation
'Hearing assistive device patients.'

Approximate time allocation for this question: 5 minutes

4. What do you think are some of the advantages or benefits of telehealth?

Free probes:
'Can you elaborate further?'
'Can anyone think of anything else?'

Approximate time allocation for this question: 10 minutes

5. What do you think are some of the disadvantages or potential concerns you may have regarding telehealth?

Free probes:
'Can you elaborate further?'
'Can anyone think of anything else?'

Approximate time allocation for this question: 10 minutes

6. Of all the aspects we've discussed today, what do you think is the most important point regarding telehealth services?

Approximate time allocation for this question: 5 minutes

Summary question

The moderator will provide a brief oral summary, and after that, this question is asked:

7. Is this an adequate summary?

Approximate time allocation for this question: 2 minutes

Closing question

The moderator then reviews the purpose of the study, and thereafter this question is asked:

8. 'Have we missed anything, or is there anything else you would like us to highlight in the research report about the use of telehealth services in the public health care system?'

That concludes our focus group session. Thank you so much for sharing your thoughts and opinions with us. A Takealot voucher for the value of R150.00 will be sent to each of you as a small token of our appreciation.
