# Supplementary material for: Perceptions of Telehealth Services for Hearing Loss in South Africa’s Public Healthcare System
Source: Int J Environ Res Public Health. 2022 Jun 24;19(13):7780. doi: 10.3390/ijerph19137780 (PMC9265507; doi:10.3390/ijerph19137780)
Supplement: Supplementary file 1 [file ijerph-19-07780-s001.zip › Table S1.pdf]

Table S1. Constructs, Items per Construct and the Corresponding Cronbach's Alpha Values

Construct name	Items / statements	Cronbach's alpha
Lack of scope from senior management and colleagues in introducing telehealth	Lack of scope from senior management in introducing telehealth	0.927
	Lack of scope from colleagues in introducing telehealth	
Risk of automated jobs resulting in no human interaction and impersonal care	The risk of making hearing care impersonal	0.789
	The risk that my job may be automated	
Internet access at worksite inadequate (unavailable, unreliable or doesn't have enough bandwidth)	Internet access at my site is unreliable	0.707
	Internet access at my site doesn't have enough bandwidth	
	Internet access at my site is unavailable	
Lack of practical training on and confidence in using telehealth	Lack of practical training on telehealth	0.690
	lack of confidence using telehealth technology	
